# The ethics and value of contact tracing apps: International insights and implications for Scotland’s COVID-19 response

**DOI:** 10.7189/jogh.10.020103

**Published:** 2020-12

**Authors:** Claudia Pagliari

**Affiliations:** Usher Institute/Edinburgh Global Health Academy, The University of Edinburgh, Edinburgh, UK

## Abstract

The COVID-19 pandemic has put health systems, economies and societies under unprecedented strain, calling for innovative approaches. Scotland’s government, like those elsewhere, is facing difficult decisions about how to deploy digital technologies and data to help contain, control and manage the disease, while also respecting citizens’ rights. This paper explores the ethical challenges presented by these methods, with particular emphasis on mobile apps associated with contact tracing.

Drawing on UK and international experiences, it examines issues such as public trust, data privacy and technology design; how changing disease threats and contextual factors can affect the balance between public benefits and risks; and the importance of transparency, accountability and stakeholder participation for the trustworthiness and good-governance of digital systems and strategies.

Analysis of recent technology debates, controversial programmes and emerging outcomes in comparable countries implementing contact tracing apps, reveals sociotechnical complexities and unexpected paradoxes that warrant further study and underlines the need for holistic, inclusive and adaptive strategies.

The paper also considers the potential role of these apps as Scotland transitions to the ‘new normal’, outlines challenges and opportunities for public engagement, and poses a set of ethical questions to inform decision-making at multiple levels, from software design to institutional governance.

## CONTEXT

The rapid escalation of COVID-19, from a small disease cluster in China in late 2019 to a global pandemic by early March 2020, took most countries off-guard and has placed extraordinary burdens on health services worldwide. Asian countries with recent experience of similar epidemics were quick to respond, implementing various measures for prevention, mitigation and control, and many Western countries have followed suit. The UK has fared less well, however, and at the time of writing had one of the world’s highest rates of infection and mortality [1]. Many factors have contributed to this, including insufficient pandemic planning, restricted supplies of personal protective equipment (PPE), lack of testing at scale and a short-lived government strategy of letting the disease spread in the hope of generating herd immunity [2].

Recent sustained decreases in new COVID-19 cases, hospital admissions and mortality rates are encouraging. However, preparations are continuing in anticipation of a possible ‘second wave’ of the virus in the autumn/winter, coinciding with the annual flu season. Ongoing vigilance will also be required to quickly identify and robustly mitigate local spikes of infection [3].

## THE CASE FOR DIGITAL CONTACT TRACING

Human contact tracing is a longstanding and proven method used in infectious disease control. Typically, once a case has been confirmed, trained personnel work with the patient to identify the places they have visited and the people they have been in close proximity to during a specified period. Efforts are made to reach those people, who are asked to self-quarantine and to contact health services if they experience symptoms. They may also be required to take a laboratory test. If all parties are successfully identified and comply with what is asked of them, the risk of onward transmission is removed.

The volume and geographic spread of COVID-19 makes this labour-intensive task extremely challenging and the UK has struggled to recruit and train sufficient numbers of contact tracers to meet the demand. For this reason, Scotland, like the other devolved UK nations and countries elsewhere, has been looking towards new technologies, including mobile phone apps, to help with this effort [4,5].

Apps associated with contact tracing vary from simple geolocation tools to multifunctional support and data collection platforms. *Proximity tracking* is a common feature, using Bluetooth Low Energy beacons to exchange encrypted ‘handshakes’ between smartphones when they are within a risk radius for a defined period of time. If a user tests positive and confirms this using the app, other users who may have been infected can be automatically notified, without revealing either party’s identity. This includes strangers, who can be impossible to reach using conventional contact tracing. Depending on the system, alerts may be accompanied by quarantine instructions or details of testing facilities. Some contact tracing apps also include *symptom reporting* tools, which, if used by enough people, can help to ‘crowdsource’ data on the incidence and spread of illness in communities, complimenting and augmenting conventional disease surveillance methods [6].

Other technologies used in contact tracing apps include satellite navigation (GPS), for tracking actual location and movements [7], and self-completed diaries or ‘check-in’ tools, which allow users to cross-reference places they have visited with public data on locations linked to confirmed cases [8].

Beyond contact tracing apps, which are the focus of this paper, technologies such as Wi-Fi and Internet of Things are also being used during COVID-19 to track peoples’ movements within buildings or city zones [4,9], while governments in some countries are harvesting citizens’ location and contact information directly from mobile network providers (see below).

## PRIVACY, RIGHTS AND POWER

Ensuring the health, safety and security of Scotland’s people is critical as we face this global pandemic yet doing so must not come at an unacceptable cost to their privacy or civil liberties.

In democratic countries during COVID-19, ethical debates surrounding the use of digital health technologies have chiefly concerned the extent to which they provide *anonymity* for individuals, *security* for their personal information, and protection for their *rights* as members of a fair and lawful society.

Critical questions also concern the *power* and *control* different actors hold over these technologies and the data they yield, which may include not only public health authorities, but also other governmental agencies (eg, police, immigration, local authorities), quasi-governmental organisations (eg, universities), third sector bodies (eg, elder care services), technology companies (eg, providers of operating systems, software, data hosting platforms) and various ‘shadow’ players (eg, health insurers, food retailers, credit reference agencies, data brokers).

The public’s acceptance of *passive* digital health surveillance tools such as thermal imaging cameras, their willingness to *actively* install and use mobile apps, and their comfort with different levels of *data* *sharing*, are influenced at least as much by their ***trust*** in these actors as in the technologies themselves.

The databases and algorithms associated with contact tracing apps have been a major focus of debate in this regard. In the case of proximity tracking, these may be primarily controlled by a government entity (*centralised*), held on users’ phones (*decentralised*) or use a hybrid of these. Since these terms will reappear in the paper, readers are advised to read the plain English summary in Appendix S1 of the **Online Supplementary Document**.

## CULTURE, SYSTEMS AND COMPLIANCE

Governments in countries such as China, South Korea, Israel and India have adopted highly privacy-invasive and coercive approaches to help control the spread of COVID-19, including precise location tracking of identifiable individuals, personal data mining, pervasive facial recognition technology and wearables for enforcing quarantine [10,11]. In general, their citizens have complied, whether through choice, conformity and a sense of collective responsibility, or because the price of not doing so includes fines, unemployment or imprisonment [12].

In contrast, the desire for freedom from government surveillance and interference is very strongly felt in Europe and privacy is a highly political issue. As such, apps that use anonymised or pseudonymised proximity logging have been favoured over those that use location tracking during COVID-19. The prospect of identifiers or proximity logs being stored on a government database has faced strong opposition, however, on the grounds that individuals could, in theory be reidentified, their social networks mapped through association, or the data otherwise misused [13]. Although governments have contested this, these concerns have led some countries to abandon their plans and change course, as with Ireland and Germany’s decisions to move from centralised to decentralised approaches [14].

Different jurisdictions *within* countries also have their own micro-cultures, as we are seeing in some European regions and in the contrasting approaches of the devolved UK governments towards contact tracing apps during COVID-19.

***England*** has been developing its COVID-19 app, commonly referred to as the NHSX app, since April and encouraged every citizen to download it after national rollout [15]. This uses Bluetooth proximity tracking but is centralised insofar as the pseudonymised [16] identity allocated to users on registration is stored on a government database, along with their IP address and a partial postcode. Digital ‘handshakes’ are kept on users’ phones unless they report coronavirus symptoms or a positive test result using the app, in which case they are shared with the NHS database and used to send anonymised alerts to at-risk users, along with advice to self-isolate. In a surprising development, on June 18^th,^ it was announced that the pilot project would cease, due to technical problems mainly affecting iPhones, which could be resolved using the new Apple-Google API [17] [see Appendix S1 in the **Online Supplementary Document**]. Although few details had been released at the time of writing, it is envisaged that the revised app will have the same look and feel, although less capability for information collection, since the API is only available to apps which use decentralised data storage [18].

***Scotland*** has so far prioritised software for assisting local professional contact tracers during the crisis [19]. The government’s ‘Test, Trace, Isolate, Support’ strategy, unveiled on May 4^th^, also included a tentative proposal from the Digital Health and Care Institute [20] for a Bluetooth-free contact tracing app, aimed at helping Covid-positive patients to report their recent encounters and daily symptoms. Data from the app would be linked with test results and health records and held centrally. The option to adopt the public-facing NHSX app at a future date was nonetheless under consideration. A separate information app has been developed by the national telehealth organisation, NHS24 [21].

***Northern Ireland*** has released a citizen-held app offering information and chatbot advice [22], but its contact tracing approach relies on traditional methods at the present time. A decentralised proximity tracking app, based on the Apple-Google API, is planned, to prioritise privacy and ease cross-border exchanges with the Irish Republic [23].

***Wales*** has hesitated on whether to adopt the NHSX app, or indeed any Bluetooth-based approach, and has instead been encouraging its citizens to use a semi-commercial third-party app, the Zoe COVID-19 Symptom Tracker, to record their symptoms and track their location. This information is shared daily with the government and NHS [24].

(NB, The above summaries are based on information available at the time of writing and the situation is constantly evolving.)

## COMPLEXITY AND TERMINOLOGY

A key challenge for citizens, policymakers and journalists seeking to make sense of contact tracing apps, is the *variety* of different approaches being proposed and the *language* used to describe them.

Discussions in the news media, tech blogs, academic papers, parliamentary committees and online social networks may refer to the *technologies* themselves (hardware, software, architectures, connectivity), their different *purposes* (eg, identifying contacts, reporting symptoms, policing quarantine, verifying immunity) or the ways in which they handle *data* (eg, centralised, decentralised). Digital contact tracing approaches can also be complex, multi-component and co-dependent, making it difficult to discuss them in isolation.

Confusion has also arisen due to the proliferation of ‘COVID-19’ apps unconnected with contact tracing, including local information tools, personal symptom checkers or advice chatbots.

The accessibility of relevant technical concepts has also hindered public communication. For example, it is common to hear Bluetooth protocols described as apps or proximity tracking described as contact tracing, whereas these are not strictly accurate. Widespread interest in the privacy models underpinning proximity tracking has compounded this confusion, by jettisoning abstract abbreviations like PEPP-PT, DP-3T, API and NHSX into the public discourse. (PEPP-PT, DP-3T, the Apple-Google API and NHSX refer to alternative proximity tracking models. NHSX refers to both an app and a UK government technology unit.)

Paradoxically, this complexity can also lead to over-simplification; for example, narratives around proximity tracking often use decentralised and centralised as synonyms for private and non-private, without considering their different models and security vulnerabilities, or those of the apps and databases they may be connected with [25,26].

## USEFULNESS AND RISK

While protecting health is a universal goal, deciding on what best serves the ‘common good’ can be a challenge, as privacy and safety are both important. Several factors need to be taken into account in order to find the right balance for digital health during COVID-19.

A key consideration is the ***usefulness*** of the technology, and thus the value trade for citizens’ privacy. Different digital architectures and changing contextual factors can affect this balance.

Centralised apps are potentially more ‘useful’, in terms of their ability to generate data for public health intelligence, research and innovation, but their capacity to do so presents a paradox, since fears over surveillance can decrease people’s willingness to use them.

Aside from privacy, there is a more fundamental issue that has, until recently, been overlooked in the hype around proximity tracking apps’ potential to automate and scale contact tracing. - *Without near-universal usage (60%+) and widespread community testing, their ability to link true cases to people they may have infected is very limited* [27]. This is why gaining enough trust and enthusiasm for these apps to be adopted and used is so critical, whilst in parallel scaling-up testing services.

In the absence of scaled testing, the UK has had to rely heavily on people with minor symptoms to self-diagnose and self-quarantine. While this puts control in the hands of the citizen, self-diagnosis is unreliable and, if used to trigger proximity alerts, is likely to generate a high number of *false positives*, unnecessarily burdening the NHS through ‘worried-well’ demands or diversion of human contact tracers. Precision will increase if the app requires a positive test result before the alert is triggered, but this requires verification by a third party - either the health authority or a server authorised by the mobile provider, theoretically raising the privacy risk.

Beyond the technology itself, the usefulness and acceptability of different digital approaches during COVID-19 also depends on the level of ***threat*** presented by the disease and the ***stage*** of the epidemic. For example, at the start of an outbreak, where few people have been affected, the greatest benefit may come from tools to augment and support traditional contact tracing. In the midst of a deadly and uncontrolled epidemic, the case for additional surveillance and data linkages will increase and citizens may be more willing to collaborate in health intelligence gathering by sharing their location or symptoms via an app. When the risk of the disease is low for most people, or the incidence is declining, less intrusive and more targeted methods will be more appropriate. As lockdowns end and people return to work and school, automated anonymised proximity logging and digital immunity passports could prove reassuring and enabling, while biometric temperature scanners might become normal in airports, hospitals or offices. On the other hand, citizens may call for such technologies to be outlawed after the pandemic is declared over, although pragmatism is likely to play a role, given the risk of further outbreaks. (NB, These are theoretical use cases, intended to illustrate the point about changing needs.)

The problem is that, in a fast-moving epidemic, governments can become so preoccupied with protecting the health system, saving lives and bringing in emergency powers, that they fail to consult with those they are trying to protect, leaving us with little ***evidence*** about what is or is not likely to be considered necessary, proportionate, acceptable or used in different situations.

## PUBLIC ENGAGEMENT AND DIGITAL INCLUSION

Understanding the needs and tolerances of different users or ‘publics’ is essential for digital projects and the history of e-government is littered with examples of failure and scandal when this was not appropriately done [28]’ While previous research on public attitudes to digital health and big data is a useful guide [29], and there is a long literature on human behaviour during disease outbreaks [30], there is relatively little evidence at the intersection between these two.

Various methods of public engagement exist, some more geared towards to *informing* citizens of new services, policies, risks or technologies, others towards *understanding* their needs, attitudes and concerns, and others aimed at *involving* them in decision-making or policy shaping. Challenges during an outbreak are that these methods can be time consuming or usually require physical meetings. Digital alternatives exist, however, some of which have already been used to seek public opinions on contact tracing apps and related innovations during COVID-19. These include online surveys [31-33], staged deliberative engagement approaches [34] and open consultations, such as the one recently undertaken by the Scottish Government [35]. So far, these have relied on small or self-selected samples, making the results difficult to reconcile and interpret.

Just as a lack of access to technology, or ***digital exclusion*** [36]*,* affects some people’s ability to benefit from online services and apps during a pandemic [37], it also affects the way in which they can be informed, consulted, engaged, or involved in decision making. This is doubly problematic, since these are often the same elderly or vulnerable groups most at risk from COVID-19 and in need of support. While the Scottish Government and some third sector bodies are making great strides with digital inclusion [38], these are unlikely to be sufficient. Analogue methods, such as telephone surveys, radio or television phone-ins, and paper-based questionnaires, are all possible ways to gather these citizens' views. In the present circumstances, however, engaging with community leaders and support groups as intermediaries may be more useful and effective.

With respect to *vulnerable groups*, so-called ‘shielding’ programmes, may disproportionately affect the digitally excluded whilst still involving them in digital communications and data sharing. Although there is little public documentation on these processes, anecdotes from some parts of the UK include personal details being shared with supermarkets by the NHS and local authorities, and third sector workers communicating about named individuals using unapproved commercial platforms like WhatsApp, neither taking place with informed consent. This sets up obvious ethical tensions between the need to protect people from hunger or harm, and the need to respect their privacy and choice. While the speed at which these initiatives had to be developed offers some excuse, and relevant third sector actors are mainly benign, action is needed to ensure that these processes have adequate governance.

## OBJECTS OF TRUST

Scotland’s strategy for public engagement should also be guided by the emerging consensus on ethical considerations for COVID-19 apps.

While there are variations in how these are being expressed in different communities of practice (eg, computing, law, social science, consumer advocacy) most include the broad themes already described, as well as concepts seen in compatible technology ethics frameworks developed in recent years; some general (eg, responsible innovation, ethical computing, privacy-by-design) and some specific to particular innovations (eg, big data, artificial intelligence, apps, facial recognition).

There are various ways of summarising these considerations and principles, to inform ethical decisions about contact tracing apps and related digital tools during the pandemic. One of these is according to the objects, entities, people or qualities requiring ***trust***, and the different questions these give rise to (**Box 1****).**

Box 1 **Objects of Trust and Ethical Questions for COVID-19 Apps.**

The **technology** – Will it work reliably? Is it safe? Is it secure? Is it private-by-design? Is it co-dependent on any other apps, databases or technologies (eg, AI) that could alter these properties? Is the software code open to scrutiny by others?

Its data **privacy policies** – Does it capture or use only the minimum necessary data? Is consent required? How anonymous is it? Is it clear who it will be shared with and for what purposes? Will it be deleted after COVID-19? Are these policies adequately explained and accessible to users?

Its **usefulness** – Is it really needed for this purpose? Does it achieve what it claims to? Is the value for citizens worth the privacy trade? Will it divert resources from more useful activities?

Its **optionality** – Are citizens free to choose whether or not to use the app, or particular features within it? If so, is this a genuine choice (eg, not being able to return to work/ school otherwise)? Is it easy to control how data is shared by opting in or out?

Its **fairness** – Could be used in inequitable or discriminatory ways? Is it disproportionately intrusive, exploitative or coercive? Are the app and its benefits accessible to all (digital inclusion)? Could it restrict people’s liberty?

The **people** driving or developing it – Are they being transparent about the project’s ambitions and scope? Do they have secondary motives or conflicting interests?

The **institutions** responsible for delivering it – Is there sufficient oversight and accountability; are there adequate processes and expectations for stakeholder involvement?

The **users** – Is it vulnerable to misuse in ways that could harm or inconvenience others?

Thus we may question the ***technology*** itself (is it reliable, safe to use, secure, private-by-design, co-dependent on riskier technologies?), its data ***privacy policies*** (does it use only the minimum necessary data, is consent required, how anonymous is it, will it be deleted when no longer required, are these policies clear to users?), its ***usefulness*** (is it necessary for this purpose, does it achieve what it claims to, will it divert resources from more useful activities, will it be decommissioned after the crisis?), its ***optionality*** (can citizens choose whether or not to use the app or particular features within it, is this a real choice or could it affect their ability to return to work, school or travel? Are users able to control how their data is shared, by opting in or out?), its ***fairness*** (is the app inclusive/accessible to all citizens, could it be used in inequitable or discriminatory ways, is it disproportionately intrusive, exploitative or coercive, does it unfairly favour certain stakeholders?), the ***people*** involved in developing it (are they being transparent about the project’s ambitions and scope, do they have secondary motives or conflicting interests?), the ***institutions*** delivering it (is there sufficient oversight and accountability; are there adequate processes and expectations for stakeholder involvement?)

These issues relate mostly to our trust in technologies and institutional actors, but trust in the ***users*** of these technologies should not be overlooked. For example, an app for reporting symptoms, test results or known contacts could be used maliciously to inconvenience others, or someone with active COVID-19 may ‘forget’ their smartphone when leaving home, to evade quarantine, thus prioritising their own freedom over others’ health [39].

Compatible ethical principles have been outlined in various recent reports on contact tracing apps, including from the UK Centre for Data Ethics and Innovation (*value, security/privacy, accountability, transparency, control)*[40], the World Health Organisation (*time-limitation; testing/evaluation; proportionality; data minimisation; use restriction; voluntariness; transparency, explainability)* [41] and Oxford Internet Institute (*necessity, proportionality, validity, time-limitation)*[42], and with overarching concerns about *power*, emphasised by the Data Ethics EU group in the context of COVID-19 [43].

Given the considerable **cost** of government technology projects, ensuring that public money is spent wisely, appropriately and fairly is also an ethical issue [44], as this can impact resources remaining for patient care. (For example, the stalled project to develop and test the NHSX app has reportedly cost £12M so far, with pundits left unsure of how this value is to carry forward to the next phase.) This issue overlaps with those around usefulness, transparency and institutional accountability in the list above, and broader frameworks for public service ethics [45].

## REGULATION AND RIGHTS

Ethics naturally intersects with regulations and laws, particularly, in the case of COVID-19 technologies, those governing **data protection** and **human rights**.

In May the UK Information Commissioners Office developed a set of data protection principles for contact tracing apps, including *transparency around purpose/design/benefits, data minimisation and anonymisation, time limitation, secure processing; user control/choice,* and *avoiding unintended privacy risks* (eg, not requiring users to leave their phones unlocked) [46].

A draft Bill on digital contact tracing apps was also put forward in May by the UK Joint Human Rights Committee, aimed at securing legislation to restrict the use or retention of data for purposes other than managing the outbreak [47]. The committee’s earlier inquiry had concluded that such an app may be useful but called for guarantees around *efficacy, proportionality, primary legislation, oversight, regular reviews and transparency* [48]*.* The proposals were rejected by the leader of the House of Commons, based on the Minister for Health’s assurance that existing legislation is sufficient [49].

Debates over contact tracing apps also have potential to intersect with anti-discrimination laws. For example, marginalised groups, such as refugees or the homeless, may lack the smartphones needed to use these apps or may have previously suffered abusive surveillance and choose not to, either way losing out on their potential benefits [50]. Advocacy groups have accused proximity apps of being “a toy for the digital elite”, due to their limited usefulness in deprived areas with overcrowded housing [51]. Higher rates of COVID-19 infection and mortality seen in some UK ethnic minorities [52] also suggests a need for more accessible apps yet capturing ethnicity-linked data poses potential risks for discrimination [53], calling for complex ethical decisions.

Broader NHS information governance controls have been slackened during COVID-19, to expedite data sharing for public health, patient care, research and innovation [54]. This has implications for the data generated by apps, insofar as this may be stored on databases, linked with other types of data, processed and repurposed, or shared within or beyond the organisation.

It is noteworthy that, on June 15^th,^, Norway was forced to close its proximity tracking app, which uses both Bluetooth and GPS, after it was judged to have breached the EU General Data Protection Regulation by collecting personal location data [55].

Legislation on notifiable diseases [56] and quarantine [57] has traditionally presented ethical tensions between public safety and individual privacy, choice or liberty. Contact tracing apps could, in theory, provide a new medium through which people may be *compelled* to reveal that they are ill or at risk, who they have spent time with and where they have been. Depending on their additional functionalities, such apps might also be used to *enforce* quarantine by monitoring users’ location or capturing proof of compliance. For example, Poland’s COVID-19 quarantine app requires users to upload a geo-tagged ‘selfie’ every 20 minutes, to prove they are still in the same place [58]. Under new rules for international travellers entering the UK [59], the introduction of similar apps is not inconceivable.

Digitisation can also create *criminal* risks, such as identity theft and fraud, as seen in a spate of text messages falsely claiming to be from contact tracers following the announcement of NHS England’s Test and Trace programme [60]. Cases of bogus COVID-19 tracker apps being used to spy on smartphone users have also been reported [61].

The broader construct of **good governance**, first developed in the context of international development, has been adapted as a way of understanding the trustworthiness of public sector health systems and the role of technologies as part of this [62]. Theories of good governance emphasise the importance of *transparency, accountability, effectiveness, fairness, participation, inclusion, sustainability* and the *rule of law*, embedded within an overarching ethic of *responsible use of power* *at all levels of institutions* [63]. As such it closely maps the proposed list of ethical considerations for COVID-19 apps.

## TRANSPARENCY AND ACCOUNTABILITY

During a crisis like COVID-19, being open about emerging technologies, models or data uses is vital, even if this involves discussing uncertainty, preliminary ideas or ‘works in progress’.

Secretive and overly-centralised decision-making can lead to mistakes in design, strategy or judging the public mood, that might have been avoided with more experience at the table. For example, earlier release of the NHSX app’s software code could have prevented embarrassing security glitches which came to light after go-live [64]. Similar issues have been raised in relation to the government’s Scientific Advisory Group for Emergencies and the data modelling which led to the unfortunate March 12^th^ decision to abandon testing and tracing [65,66].

Lack of transparency can also leave observers in a constant state of *uncertainty* about the solutions being developed. This is evident in continuing ambiguities around the privacy architectures and functionality of government apps, and their relationship with national contact tracing initiatives [67]. Likewise, it may result in tentative ideas or proposals being misinterpreted as concrete strategies or developments, as seen in optimistic media reports about digital ‘immunity passports’ during April, based on assumptions about antibody tests that were later questioned by the World Health Organisation [68]. (NHSX recently revisited this idea, linked to a proposed facial recognition feature [69].)

Significantly, it leaves an information void that can be filled with rumours and speculation, which can be reputationally damaging and strategically disruptive. These may spill over to damage routine services or cause unnecessary alarm to citizens [70].

The influence of transparency and accountability can be *indirect*, as recently seen in England, where public anger at a political figure who flouted lockdown rules, apparently with no consequences, affected citizens’ intentions to install the COVID-19 app as part of the Test and Trace strategy [71].

With the UK perilously close to a ‘second surge’, it is vital that we can rely on people to notify the health service if they fall ill, or to self-quarantine if they discover they may have been exposed. Apps have potential to facilitate this, whether through proximity tracking, symptom reporting or even simple health messaging, but when trust in leadership is diminished, so is the *social capital* that is essential for success.

## MISSION CREEP AND BOUNDARY LINES

In addition to concern about the surveillance state, the prospect of *mission creep* has been frequently expressed during COVID-19 [72]. This refers to the possibility that technologies and data flows implemented for managing the pandemic will be used for other governmental purposes - such as policing or designing ‘nudge’ techniques - as well as for unspecified academic research or commercial innovation.

The related term ‘function creep’ has also been used, both synonymously with the above and to describe the stealthy insertion of additional app features that could erode privacy [73].

These concerns have been exacerbated by the participation of private technology companies in the NHS COVID-19 response, particularly those associated with the policing or defence sectors, or with controversial track records in personal data harvesting [74]. Fears have been raised about the security of the NHS data which is being hosted, mined or linked by these companies, and whether this may be used to generate consumer profiles, proprietary artificial intelligence algorithms or new vendor lock-ins [75]. The awarding of lucrative government contracts to some of these companies, outside normal procurement channels, has also raised concerns, given the high stakes for both privacy and public finance [76].

NHSX has sought to allay fears over mission creep by issuing statements about data security and usage restrictions, but not all of these are consistent. For example, when questioned in a parliamentary evidence session, the director revealed that in addition to supporting ‘anonymous’ proximity tracking, the system would seek users’ consent to capture identifiable data that could be re-used for future research and analytics [77].

Another influential set of players in COVID-19 is the *research community*, including universities, government research and innovation sponsors, independent foundations and their private partnerships. These are highly motivated to acquire and use patient data and have previously been effective in lobbying for special allowances under the General Data Protection Regulation [78]. While there are certainly many ethical uses of data to benefit patient health, extensive government investment in health data research, coupled with incentives for academics and medics to spin-off their data-driven innovations, has created new challenges for differentiating public and commercial activities. With clinical data scientists now occupying positions of influence within government, on research funding councils and on key COVID-19 decision making groups, separating these agendas is increasingly difficult.

The situation with COVID-19 apps in Scotland illustrates some of these difficulties around scope and boundaries. As already noted, the approach summarised in the Scottish Government’s ‘Test, Trace, Isolate and Support’ strategy [20] prioritises professional contact tracing over mass Bluetooth tracking, and as such may be seen as less prone to privacy violations or mission creep. However, in parallel, Scotland’s medical researchers are encouraging citizens to use the semi-commercial Zoe app which, in addition to allowing users to track their own symptoms, requests access to this information along with location, demographic information and any test results [79]. These are relayed to the SAIL databank, where they can be linked with other health records and accessed by researchers via the HDRUK Innovation Gateway. Despite its requirement for personal information, nearly 3 million people have downloaded this tool and researchers are already publishing analyses of the data in prestigious medical journals. Although the generation of evidence may be framed as a ‘public good’ [80] - for example data collected using the Zoe app has verified that taste and smell are indicators of infection [81] - this data mining runs contrary to the spirit of other government communications and could lead to confusion. Moreover, it creates a ‘side door’ to the sort of data that has proven so controversial when governments have tried to integrate this into their own contact tracing apps. With the NHS now co-funding the Zoe tool, and able to access it via SAIL/HDRUK, it is conceivable that similar symptom reporting features in the NHSX app will be de-prioritised.

This example, amongst others, shows the importance of transparency for effective accountability as well as the difficulties this can present. For example, Zoe and many other apps that are now being encouraged to share data with the NHS under the ‘Oasis’ scheme [82], require explicit ***consent*** from users, so there may be no regulatory impediments under GDPR, and it might be presumed that research based on the data has been subjected to appropriate ethics review and privacy impact assessments. However, it cannot be guaranteed that users recognise the full implications of what they are consenting to share. More importantly it raises questions about *honesty* in communicating with the public, if the government’s dominant privacy assurances concern contact-tracing tools, while significantly more user data is being exchanged by parallel apps and data centres co-sponsored by the same government.

Misalignments between different authorities governing the usage of digital health data and technologies can also present challenges, requiring action at the level of government, health organisations, university ethics committees, medical device regulators and more, with no overarching oversight, resulting in gaps and vulnerabilities [83,84].

## INSIGHTS FROM COMPARABLE COUNTRIES

### The privacy-trust-participation enigma

The ethical narrative around COVID-19 apps has been dominated by a desire to ‘engineer-in’ privacy and security, based on the assumption that these are keystones for trust and usage. While growing interdisciplinary involvement has gradually opened up broader dialogues around rights and proportionality, privacy remains a central theme.

Experiences from countries similar to Scotland suggest that the relationship between these factors and citizens’ willingness to participate is complex, however. Even where key ‘ethical’ requirements are satisfied, adoption is not guaranteed, while tools that may be regarded as intrusive have, in some cases, faced surprisingly little resistance.

Singapore is a small democratic country with a population roughly the size of Scotland’s. It enjoys high levels of public trust in government [85], high compliance with rules, high levels of digital inclusion and its COVID-19 app ‘TraceTogether’ uses the (semi-) decentralised model favoured by privacy professionals. Despite all of these factors, less than a quarter of the population has downloaded the app, due both to privacy concerns and because the current version depletes mobile phone battery life [86]. While updates to the iOS and Android operating systems may address the latter problem, it remains to be seen whether rates of uptake increase.

Australia has suffered from public mistrust in digital health for some time, most recently with the My Health Record initiative [87]. The government has therefore gone to great lengths to assure citizens of the security and privacy of its COVIDSafe app, which is based on the Singapore model. This includes strict legislation to prevent misuse of the data [88]. Between April and June the app had been downloaded by nearly half of all citizens. Interestingly, the results of a recent survey suggest that those who had not done so were influenced less by privacy concerns than by the perception that COVID-19 is not severe enough to warrant it, illustrating the earlier point about risk perceptions [89].

New Zealand’s government benefits from high public trust, which has increased during COVID-19 [90]. Its voluntary, Bluetooth-free, Covid Tracer app enables users to scan QR codes, located in public buildings or businesses, to create a personal digital diary. If they test positive for COVID-19, the public health authority will request permission to add this information to a database of time-stamped locations. This can be anonymised and broadcast to registered phones, and if a location match is found the recipient will be alerted. Despite initial surveys suggesting that most citizens would download the app, by late May only c.7% had done so [91]. This suggests that while prioritising user control is ethical it may in some cases limit, rather than encourage, uptake [92]. (A proposal to integrate Bluetooth tracking raised privacy concerns and the government has decided to stick with the current choice-based system).

Iceland is another small country which benefits from high levels of mutual trust, to such an extent that much of the population has willingly been genotyped by a company working with the government [93]. Its national Covid app uses GPS location tracking, which is far less private than the Bluetooth proximity method. Nevertheless, by early May it was the country with the highest percentage of people using such an app, illustrating the power of national trust and collectivism [94].

### The value proposition: real or perceived

The cultural factors affecting trust are clearly important in explaining these nuances and inconsistencies, but a bigger finger points to the role of *necessity* in people’s willingness to use COVID-19 apps. As discussed in a previous section, the value proposition for COVID-19 apps and other technologies can change at different stages of a pandemic, which affects both what governments are willing to invest in and what citizens are willing to accept.

In Singapore the virus has been largely contained, with nearly all cases now being migrant workers quarantined in secure dormitories [95]. While the latter raises difficult ethical questions about discrimination, inequalities and effects on liberty, there is a *sense that for most people the risk is low, which is affecting their motivation* *to engage*, particularly given the privacy and battery life issues mentioned earlier. Paradoxically this – along with concern over digital exclusion - prompted the government to take what might be regarded as the more draconian step of mandating Bluetooth wristbands for ‘every resident’. After a public outcry this has been scaled back to only people in quarantine, echoing a system used in Hong Kong [96].

Australia has been relatively successful in persuading the public to use COVIDSafe in the month since its launch but, as already noted, a large section of the population remains to be convinced of its necessity. More significantly, as of mid-June, only one case unknown to government contact tracers had been identified via the app and it has gone from being seen as “*vital to almost useless”* [97]. Problems with Bluetooth connectivity have also created difficulties [98].

By June 1^st^ Iceland had defeated the virus, aided by a combination of contact tracing and the DEecode genetics company turning its labs over to COVID-19 testing [99]. Despite good public engagement with the app, public health officials have judged it *“more or less… useless”* for identifying cases missed by conventional contact tracing, echoing Australia’s experience [100].

New Zealand’s Covid Tracer app was released two weeks after the country had brought the disease under control with aggressive testing, tracing and travel restrictions [101]. Despite this, the government has continued to promote the app, placing the greatest emphasis on its benefits for *prevention* at this time. After nearly a month with no new cases, a quarantine breach by two Covid-positive visitors from the UK on June 16th prompted a flurry of contact tracing, as well as military involvement in enforcement. This event has emphasised the country’s need to remain vigilant and may strengthen the public’s willingness to participate [102].

As these examples show, achieving success with contact tracing apps involves a complex mix of social, biomedical, political and digital factors, not all of which are easy to anticipate. It also illustrates why a static approach to apps in a rapidly evolving environment is unwise. Revisiting the changing value proposition at regular intervals is important, as changing circumstances can alter the potential benefits these tools offer. This may require the ethical ‘sunsetting’ or ‘hibernation’ of apps, or particular features within them, that are no longer useful or necessary; likewise, closing data channels that may have been vital for health intelligence during a pandemic but are disproportionate when threat levels drop. The ability to do this in a dynamic, responsive and transparent way is vital.

## FINDING THE ETHICAL PATH FOR SCOTLAND

The Scottish Government is having to make rapid and important decisions about how to respond digitally to COVID-19. This requires carefully balancing respect for citizens’ rights with a desire to use technology and data to support disease surveillance and control, health service delivery, research and innovation.

As some of the examples discussed here illustrate, public trust is critical for democracies wishing to implement contact tracing apps but can be easily damaged if their privacy, usefulness, resilience, accuracy or security is doubted, or if institutions are perceived to have overstepped their ‘social license’ for surveillance or data harvesting.

Many of these issues can be seen in the concerns about lack of transparency, mission creep and business interests that have dogged England’s NHSX during COVID-19. Scotland is also at risk of such accusations and efforts are needed to ensure that any future app, the data it yields and the uses to which this is put are proportionate, explainable, acceptable and accountable.

Understanding the ethical challenges of COVID-19 apps is impossible without also taking account of the wider digital ecosystem in which they sit. As described in this paper, apps vary in the breadth of functions they offer, their co-dependence on digital intermediaries, whether they work with or independently of government, and the propositions they present for privacy, consent and choice. Proximity tracking apps may also have features for symptom reporting, identity verification, immunity passporting or quarantine enforcement. They may use precision location tracking, automated anonymised monitoring, facial recognition technology, or feed databases that use artificial intelligence to process the information. These nuances present overlapping and unique ethical challenges.

In Scotland, as in England, discussions about these apps are taking place alongside more ambitious plans to create national intelligence platforms, which will provide a means of accessing, linking and gleaning insights from the public sector's vast data assets, and by extension its people. Lessons from elsewhere reveal how the purposes of COVID-19 apps can flip from direct public health applications to enriching large scale databases for secondary uses. Avoiding data opportunism requires robust and ethical governance, as well as honest public dialogue, to ensure that such proposals are clearly understood and adequately negotiated. Given the close parallels with activities in England, care should be taken to ensure that Scotland learns from its mistakes [103].

In the debate over the ethics of centralised versus decentralised proximity tracking, the latter has gained the upper hand; being seen as less prone to privacy violations or mission creep, and governments across Europe are moving to the model favoured by Apple and Google. While this is arguably a victory for privacy campaigners, in practice these decisions are often being taken because of the technical advantages the API offers for connectivity, sensitivity, accuracy and battery life, as seen in NHS England’s reluctant conversion. Paradoxically, this raises new and unexpected ethical dilemmas, with commentators now starting to question the appropriateness of allowing two global technology giants to force the hands of elected governments [104], whether proposals to embed proximity tracking in the iOS and Android operating systems could have future implications for choice, surveillance or commercial advantage [105], whether the campaign is being used to sanitize Google’s reputation for personal data mining [106], and if it will reduce governments’ ability to fight the pandemic by restricting the types of data they may potentially collect or use [107].

Whether it will be possible for England to create a hybrid version of the NHSX app under Apple and Google’s terms of use is still unclear. It also remains to be seen whether this will be delivered before the predicted second wave [108], or how resilient, acceptable and inclusive it will be. Either way a semi-decentralised app is unlikely to generate the volume or type of data originally envisaged. The estimated £12M spent so far, without a viable product, is testament to the costs of losing public trust [109]. This was arguably exacerbated by public perceptions of the organisation’s commercial partners, despite assurances that they would have no access to the data. It also shows the cost of failing to listen to outside advice, despite technical obstacles having been flagged at an early stage [110]. Nonetheless, there is probably some truth in The Minister for Health’s assertion that Apple could have provided a workaround, albeit one that would not have satisfied all privacy concerns [111].

Experience from Germany, which has recently flipped from a centralised model to one using the Apple-Google API, is both encouraging and offers a note of caution. Launched on June 16^th^, the Corona-Warn app was successfully downloaded by 6.5 million users in the space of 24 hours, with the numbers now having nearly doubled. However, the country’s data protection commissioner immediately objected to the fact that recipients of alerts are asked to phone the contact tracing service and give their details [112]. Unlike the UK, Germany was already well ahead with its human contact tracing programme by the time the app launched, which may increase its chance of success.

The ethical proposition for apps is closely bound up with their value proposition. Despite huge global efforts to develop these tools, early experiences point to the awkward possibility that they may offer little benefit, relative to their cost and potential privacy risks, at least as far as identifying unknown cases is concerned. As such, the techno-solutionist narratives that have dominated discussions around proximity tracking apps are subsiding in favour of seeing these as useful adjuncts to traditional contact tracing [113].

While this is a good argument for scaling back these projects and supports Scotland’s decision to prioritise professional contact tracing at the present time, *discounting these tools may be premature,* given the threat of a major second wave, the incidence of localised outbreaks, and the ethics of failing to protect citizens from disease or death. Restarting schools [114] and the economy [115] are also ethical aspirations, given the negative societal side-effects of the lockdown.

The clash between tech-optimism and public health reality is largely attributable to a failure to recognise the importance of mass participation and community testing for contact tracing apps to be useful. Wishful thinking was not only the domain of technologists; these apps were also central to early deliberations by the government’s scientific advisors [116]. *However, with testing in the UK now finally scaling, public awareness high, and experience having clarified key technological and privacy requirements, a robust, useful and ethically acceptable COVID-19 app for Scotland is now theoretically possible*.

Despite efforts to recruit an ‘army’ of new contact tracers [117], there may still be value to be gained from these apps, and the various features that can be included within them. For example, semi-automated ‘check-in’ tools could prove more convenient and acceptable for café customers than writing-down their names and addresses, as required under new legislation [118].

This paper focuses on government technologies and strategies, which are central to the management of public health emergencies. However, it is essential to note that businesses, research labs and enterprising citizens are also developing COVID-19 apps, often with far less consideration of their ethical, privacy or security implications [119]. Although Apple and Google’s rules, restricting API access to one bona fide app per country, should help to avoid the proliferation and misuse of proximity tracking, plenty of independent apps are harvesting personal data on symptoms, location, feelings and contacts. Some of these are being brought within the purview of governments, such as through the Oasis scheme mentioned earlier, potentially creating alternative channels for public health intelligence, which will require ethical and regulatory scrutiny.

The experiences of other democratic countries and the devolved UK nations provide insights into some of the complexities involved in finding the right approach for COVID-19 apps. For example, while citizens’ privacy and trust in government are vital for acceptance, they are no guarantee of adoption, use or effectiveness, which are also likely to be influenced by risk, necessity and a sense of collective purpose. Likewise, Iceland’s experience suggests that centralised approaches may be acceptable if there is a high level of societal trust, users’ privacy, choices and rights are adequately protected, and there is robust institutional governance to avoid mission creep or exploitative data practices. *As such,* *choosing one or the other approach is partly about deciding whether to commit to building public trust or outsource it to technology, although a blend of the two is ideal.*

These examples also illustrate the importance of being *critical* and *realistic* when making decisions about digital health investments, and *pragmatic* and *flexible* enough to adapt these to changing circumstances. This includes knowing when to decommission apps and turn off data flows when they are no longer necessary or proportionate, as well as how to make best use of them while they are.

With Scotland aspiring to be an *ethical digital nation* [120], wisely managing such dilemmas to best serve the public interest requires a commitment to earning citizens’ trust through effective and inclusive engagement, transparency and robust accountability.

The relative success of Germany and New Zealand in motivating public participation hints at the role of trusted leadership, with both premiers having been widely commended for their integrity during the pandemic. Scotland’s leaders also have an opportunity to harness the social capital they have accrued during this episode, to encourage app usage, but this will need to be grounded in trustworthy systems and strategies if it is not to backfire.

## CONCLUSIONS

Contact tracing apps have been the subject of considerable innovation and debate in the UK and internationally during recent months. A great deal has been learned about their limitations and ethical risks, and about the social, cultural, technical and political factors that can influence their acceptability, use and effectiveness. Despite their challenges, contact-tracing apps may yet have a role to play in Scotland’s response to COVID-19, alongside conventional methods, as the country responds to continued viral threats, while seeking to ‘recover and adjust to the new normal’ [3], build a ‘resilient wellbeing economy’ [115], and encourage ‘social renewal’ [121]. As a microcosm of the wider digital- and data landscape, this also presents an opportunity to demonstrate an ethically robust and inclusive approach to innovation in Scotland. It is hoped that the questions, examples and analysis in this paper can prompt critical discussions about digital ethics within and across relevant government departments, by leaders overseeing portfolios in which these apps are likely to sit, and by the wider community of stakeholders and citizens analysing, contemplating or adopting these technologies.

## Additional material

Online Supplementary Document

## References

[1] Burn-Murdoch J, Giles C. UK suffers second highest death rate from Coronavirus. Financial Times. 28 May 2020. Available: https://www.ft.com/content/6b4c784e-c259-4ca4-9a82-648ffde71bf0. Accessed: 1 July 2020.

[2] Matthews O Britain drops its go-alone approach to coronavirus. Foreign Policy. 2020;17 Available: https://foreignpolicy.com/2020/03/17/britain-uk-coronavirus-response-johnson-drops-go-it-alone/. Accessed: 1 July 2020.

[3] Minister F. Deputy FM. Coronavirus (COVID-19): framework for decision-making. Scottish Government. 26 April 2020. Available: https://www.gov.scot/publications/coronavirus-covid-19-framework-decision-making/pages/9/. Accessed: 1 July 2020.

[4] (Linked report) Buchanan B, Imran M, Pagliari C, Pell J, Rimpilainen S. Use of participatory apps in contact tracing: options and implications for public health, privacy and trust. Glasgow: Scottish Digital Health and Care Institute; 2020.

[5] Tibbetts JH Digital technologies aim to accelerate contact tracing. BioScience. 2020biaa071. Available: https://academic.oup.com/bioscience/article/doi/10.1093/biosci/biaa071/5864935. Accessed: 1 July 2020. 10.1093/biosci/biaa071 PMC7337627 34191865

[6] Pagliari C Vijaykumar S Digital Participatory Disease Surveillance and the Zika Crisis. PLoS Negl Trop Dis. 2016;10:e0004795. 10.1371/journal.pntd.0004795 27294787 PMC4905668

[7] O’Neill PH, Ryan-Mosley T, Johnson B. A flood of coronavirus apps are tracking us, now it’s time to keep track of them. MIT Technology Review. 7 May 2020. Available: https://www.technologyreview.com/2020/05/07/1000961/launching-mittr-covid-tracing-tracker/. Accessed: 1 July 2020.

[8] Harb R. New Zealand releases Bluetooth-free COVID-19 tracing app. The Register. 20 May 2020. Available: https://www.theregister.com/2020/05/20/new_zealand_scaled_back_digital/. Accessed: 1 July 2020.

[9] Censis News. IoT Scotland: Free access to address covid-19 challenges. April 16, 2020. Available: https://censis.org.uk/2020/04/16/iot-scotland-free-access-to-address-covid-19-challenges/. Accessed: 1 July 2020.

[10] Kim N. ‘More scary than coronavirus’: South Korea’s health alerts expose private lives. Guardian. 6 March 2020. Available: https://www.theguardian.com/world/2020/mar/06/more-scary-than-coronavirus-south-koreas-health-alerts-expose-private-lives. Accessed: 1 July 2020.

[11] Kharpal A. Use of surveillance to fight coronavirus raises concerns about government power after pandemic ends. CNBC. 26 March 2020. Available: https://www.cnbc.com/2020/03/27/coronavirus-surveillance-used-by-governments-to-fight-pandemic-privacy-concerns.htm. Accessed: 1 July 2020.

[12] Ellis-Peterson H. India’s Covid-19 app fuels worries over authoritarianism and surveillance. The Guardian. 4 May 2020. Available: https://www.theguardian.com/world/2020/may/04/how-safe-is-it-really-privacy-fears-over-india-coronavirus-app. Accessed: 1 July 2020.

[13] Academics voice concerns about UK contact-tracing app plans. Engineering & Technology. 30 April 2020. Available: https://eandt.theiet.org/content/articles/2020/04/academics-voice-concerns-about-uk-contact-tracing-app-plans/. Accessed: 1 July 2020.

[14] Abboud L, Miller J, Espinoza J. How Europe splintered over contact tracing apps. Financial Time. 10 May 2020. Available: https://www.ft.com/content/7416269b-0477-4a29-815d-7e4ee8100c10. Accessed: 1 July 2020.

[15] Griffin A. Public urged to download coronavirus contact tracing app amid questions over privacy and security. Independent. 4 May 2020. Available: https://www.independent.co.uk/life-style/gadgets-and-tech/news/coronavirus-app-nhs-contact-tracing-download-privacy-security-a9498531.html. Accessed: 1 July 2020.

[16] Burgess M. Just how anonymous is the NHS Covid-19 contact tracing app? Wired. 12 May 2020. Available: https://www.wired.co.uk/article/nhs-covid-app-data-anonymous. Accessed: 1 July 2020.

[17] Department of Health and Social Care. Next phase of coronavirus (COVID-19) app announced. 18 June 2020. Available: https://www.gov.uk/government/news/next-phase-of-nhs-coronavirus-covid-19-app-announced. Accessed: 1 July 2020.

[18] Chowdry H, et al. NHS Track and Trace all: how will it work and when can you download it. Telegraph. 17 June 2020. Available: https://www.telegraph.co.uk/technology/2020/06/17/nhs-app-trace-track-coronavirus-download/. Accessed: 1 July 2020.

[19] Downey A. Three Scottish Trusts trial Covd-19 contact-tracing software. Digital Health Networks. 20 May 2020. Available: https://www.digitalhealth.net/2020/05/three-scottish-trusts-trial-covid-19-contact-tracing-software/. Accessed: 1 July 2020.

[20] Scottish Government. Coronavirus (COVID-19): test, trace, isolate, support strategy. 4 May 2020. Available: https://www.gov.scot/publications/coronavirus-covid-19-test-trace-isolate-support/pages/6/. Accessed: 1 July 2020.

[21] Inform NHS. NHS24 Coronavirus (COVD-19) App. Available: https://www.nhsinform.scot/care-support-and-rights/tools-and-apps/nhs-24-coronavirus-covid-19-app. Accessed: 1 July 2020.

[22] Department of Health. Dedicated Northern Ireland covid-19 app launched. Northern Ireland Government March 27 2020. Available: https://www.health-ni.gov.uk/news/dedicated-northern-ireland-covid-19-app-launched. Accessed: 1 July 2020.

[23] Manthorpe R. Northern Ireland rejects UK’s COVID-19 contact tracing app. Sky News. 21 May 2020. Available: https://news.sky.com/story/coronavirus-northern-ireland-rejects-uks-covid-19-contact-tracing-app-11992232. Accessed: 1 July 2020.

[24] Hayward W. New app created to track coronavirus in Wales. Wales Online. 11 April 2020. Available: https://www.walesonline.co.uk/news/health/coronavirus-covid-symptoms-app-phone-18075865. Accessed: 1 July 2020.

[25] Hoepman JJ. Stop the Google and Apple contact tracing platform or be ready to ditch your smartphone. (Blog). 11 April 2020. Available: https://blog.xot.nl/2020/04/11/stop-the-apple-and-google-contact-tracing-platform-or-be-ready-to-ditch-your-smartphone/. Accessed: 1 July 2020.

[26] Bengio Y Ippolito D Janda R Jarvie M Prud'homme B Rousseau J-F Inherent privacy limitations of decentralized contact tracing apps. J Am Med Inform Assoc. 2020;ocaa153. Online ahead of print. 10.1093/jamia/ocaa153 32584990 PMC7337846

[27] Big Data Institute. Digital contact tracing can slow or even or even stop coronavirus transmission and ease us out of lockdown. University of Oxford. Available: https://www.bdi.ox.ac.uk/news/digital-contact-tracing-can-slow-or-even-stop-coronavirus-transmission-and-ease-us-out-of-lockdown. Accessed: 1 July 2020.

[28] Sterckx S Rakoc V Cockbain J Borry P “You hoped we would sleepwalk into accepting the collection of our data”: controversies surrounding the UK care.data scheme and their wider relevance for biomedical research. Med Health Care Philos. 2016;19:177-90. 10.1007/s11019-015-9661-6 26280642 PMC4880636

[29] Aitken M de St Jorre J Pagliari C Jepson R Cunningham-Burley S Public responses to the sharing and linkage of health data for research purposes: a systematic review. BMC Med Ethics. 2016;17:73. 10.1186/s12910-016-0153-x 27832780 PMC5103425

[30] Markel H. Contemplating pandemics – the role of historic enquiry in developing pandemic mitigation strategies for the twenty first century. In Ethical and Legal Considerations in Mitigating Pandemic Disease. Washington (DC): National Academies Press (US); 2007.

[31] The Conversation. Coronavirus: survey reveals what the public wants from a contact tracing app. 15 May 2020. Available: https://theconversation.com/coronavirus-survey-reveals-what-the-public-wants-from-a-contact-tracing-app-138574. Accessed: 1 July 2020.

[32] Crouch H. BCS survey reveals contact tracing app skepticism. Digital Health Networks. 26 May 2020. Available: https://www.digitalhealth.net/2020/05/bcs-survey-contact-tracing-app/. Accessed: 1 July 2020.

[33] Health Foundation. Contact tracing threatens to exacerbate unequal risk of COVID-19. 3 June 2020. Available: https://www.health.org.uk/news-and-comment/news/contact-tracing-app-threatens-to-exacerbate-unequal-risk-of-covid-19. Accessed: 1 July 2020

[34] McKeon A, Patel R, Burall S, Bradley J. Rapid online deliberation on Covid-19 technologies. Ada Lovelace Institute (undated). Available: https://www.adalovelaceinstitute.org/our-work/rethinking-data/rapid-online-deliberation-on-covid-19-technologies/. Accessed: 1 July 2020.

[35] Scottish Government. Coronavirus (Covid-19) Framework for decision making. Overview of Public Engagement. 21 May 2020. Available: https://www.gov.scot/publications/coronavirus-covid-19-framework-decision-making-overview-public-engagement/pages/3/. Accessed: 1 July 2020.

[36] Disconnected. Understanding digital inclusion and improving access. Citizens Advice Scotland. 18 February 2020. Available: https://www.cas.org.uk/system/files/publications/cas_disconnected_report.pdf. Accessed: 1 July 2020.

[37] Contact-tracing app risks widening digital divide, MPs told. Express and Star. 15 May 2015. Available: https://www.expressandstar.com/news/uk-news/2020/05/15/contact-tracing-app-risks-widening-digital-and-social-divide-mps-told/. Accessed: 1 July 2020.

[38] Slater A. No One Left Behind Digital Scotland: COVID-19. SCVO. 19 March 2020. Available: https://scvo.org.uk/p/36175/2020/03/19/no-one-left-behind-digital-scotland-covid-19. Accessed: 1 July 2020.

[39] Dearden L. New NHS contact tracing app vulnerable to ‘malicious false alerts’ warn expert. Independent. 5 May 2020. Available: https://www.independent.co.uk/news/uk/home-news/coronavirus-app-nhs-contact-tracing-cyber-attack-hack-a9500401.html. Accessed: 1 July 2020.

[40] Cannicott S. The ethics of contact tracing apps: international perspectives. Centre for Data Ethics and Innovation, Blog. 12 May 2020. Available: https://cdei.blog.gov.uk/2020/05/12/the-ethics-of-contact-tracing-apps-international-perspectives/. Accessed: 1 July 2020.

[41] Ethical considerations to guild the use of digital proximity tracking technologies for COVID-19 contact tracing. Interim guidance. World Health Organization. 28 May 2020. Available: https://www.who.int/publications/i/item/WHO-2019-nCoV-Ethics_Contact_tracing_apps-2020.1 Accessed: 1 July 2020.

[42] Morley J Cowls J Taddeo M Floridi L Ethical guidelines for COVID-19 contact tracing apps. Nature. 2020;582:29-31. 10.1038/d41586-020-01578-0 32467596

[43] DataEthics UK Contact tracing apps are not just a privacy tech issue it's a question of power. 20 May 2020. Available: https://dataethics.eu/contact-tracing-apps-are-not-just-a-privacy-tech-issue-its-a-question-of-power/. Accessed: 1 July 2020.

[44] Pagliari C. The risks of basing digital health strategy on industry hype and alluring prototypes. ICT&Health. 3 April 2019. Available: https://www.ictandhealth.com/news/the-risks-of-basing-digital-health-strategy-on-industry-hype-and-alluring-prototypes/. Accessed: 1 July 2020.

[45] Committee on Standards in Public Life. Ethical standards for providers of public services. UK Government. June 2014. Available: https://assets.publishing.service.gov.uk/government/uploads/system/uploads/attachment_data/file/336942/CSPL_EthicalStandards_web.pdf. Accessed: 1 July 2020.

[46] UK Information Commissioner’s Office. COVID-19 contact tracing: data protection expectations on app development. May 2020. Available: https://ico.org.uk/media/for-organisations/documents/2617676/ico-contact-tracing-recommendations.pdf. Accessed: 1 July 2020.

[47] Joint Human Rights Committee. Committee drafts Bill on COVID-19 contact tracing app. UK Parliament. 15 May 2020. Available: https://www.parliament.uk/business/committees/committees-a-z/joint-select/human-rights-committee/news-parliament-2017/covid-contact-tracing-app-draft-bill-19-21/. Accessed: 1 July 2020.

[48] Joint Select Committee. Report on the contact tracing app published. UK Parliament. 7 May 2020. Available: https://www.parliament.uk/business/committees/committees-a-z/joint-select/human-rights-committee/news-parliament-2017/covid-19-contact-tracing-app-report-published-19-21/. Accessed: 1 July 2020.

[49] LexisNexis. Data protection concerns expressed for contact tracing amid coronavirus (Covid-19) LNB News. 1 June 2020. Available: https://www.lexisnexis.co.uk/blog/covid-19/data-protection-concerns-expressed-for-contact-tracing-amid-coronavirus-(covid-19). Accessed: 1 July 2020.

[50] Human Rights Watch. Covid-19 apps pose serious human rights risks. 13 May 2020. Available: https://www.hrw.org/news/2020/05/13/covid-19-apps-pose-serious-human-rights-risks. Accessed: 1 July 2020.

[51] Reintjes R Lessons in contact tracing from Germany. BMJ. 2020;369:m2522. 10.1136/bmj.m2522 32586833

[52] Public Health England. Disparities in the risk and outcomes of Covid-19. Crown Copyright. June 2020. Available: https://assets.publishing.service.gov.uk/government/uploads/system/uploads/attachment_data/file/892085/disparities_review.pdf. Accessed: 1 July 2020.

[53] Tapper J. Minorities more at risk from COVID-19 because of racism, says report. Guardian. 13 June 2020. Available: https://www.theguardian.com/inequality/2020/jun/13/leaked-report-says-racism-and-inequality-increase-covid-19-risk-for-minorities. Accessed: 1 July 2020.

[54] Scottish Government eHealth Division. Covid-19 Information Governance Advice. Undated. https://www.informationgovernance.scot.nhs.uk/covid-19-information-governance-advice/. Accessed: 1 July 2020.

[55] Hoeksma J Norway forced to backtrack on mass surveillance track and trace app. Digit Health. 2020;18 Available: https://www.digitalhealth.net/2020/06/norway-track-and-trace-app/. Accessed: 1 July 2020.

[56] UK Government. Public Health Control of Disease Act 1984 via Wikipedia. Available: https://en.wikipedia.org/wiki/Public_Health_(Control_of_Disease)_Act_1984. Accessed: 1 July 2020.

[57] Milford A. The legal basis for quarantine. Kingsley Napley. 6 June 2020. Available: https://www.kingsleynapley.co.uk/insights/blogs/criminal-law-blog/covid-19-the-legal-basis-for-quarantine. Accessed: 1 July 2020.

[58] Chen C. Poland’s Covid-19 selfie app raises privacy questions. Privacy News Online. 25 March 2020. Available: https://www.privateinternetaccess.com/blog/polands-covid-19-selfie-app-raises-privacy-questions-will-everyone-eventually-be-tracked/. Accessed: 1 July 2020.

[59] Greig F. UK quarantine rules explained. Scotsman. 9 June 2020. Available: https://www.scotsman.com/news/uk-news/uk-quarantine-rules-explained-14-day-self-isolation-period-after-travel-explained-following-its-launch-2849322. Accessed: 1 July 2020.

[60] Citizens’ Advice Scotland. Coronavirus scams. Undated. Available: https://www.citizensadvice.org.uk/scotland/consumer/scams/common-scams-s1/. Accessed: 1 July 2020.

[61] Brewster T Coronavirus scam alert. Covid-19 map malware can spy on you through your android microphone and camera. Forbes. 2020;18 Available: https://www.forbes.com/sites/thomasbrewster/2020/03/18/coronavirus-scam-alert-covid-19-map-malware-can-spy-on-you-through-your-android-microphone-and-camera/. Accessed: 1 July , 2020.

[62] Holeman I Cookson T Pagliari C Digital technology for health sector governance in low- and middle-income countries: scoping review. J Glob Health. 2016;6:020408. 10.7189/jogh.06.020408 27648255 PMC5017033

[63] Good Governance and the Rule of Law. United States Council for International Business. January 2015. Available: https://www.uscib.org/docs/Governance%20and%20the%20Rule%20of%20Law.pdf. Accessed: 1 July 2020.

[64] Culnane C, Teague V. Security analysis of the NHS COVID-19 App. StateofIT. 19 May 2012. Available: https://stateofit.com/UKContactTracing/. Accessed: 1 July 2020.

[65] Pagliari C. Digital health and pandemics: what Covid-19 reveals about the challenges. ICT&Health. 18 Mary 2020. Available: https://www.ictandhealth.com/news/digital-health-and-pandemics-what-covid-19-reveals-about-the-challenges/. Accessed: 1 July 2020.

[66] Weinberg N, Pagliari C. Covid-19 reveals the need to review the transparency and independence of scientific advice. UK Constitutional Law Association. 16 June 2020. Available: https://ukconstitutionallaw.org/2020/06/16/nyasha-weinberg-and-claudia-pagliari-covid-19-reveals-the-need-to-review-the-transparency-and-independence-of-scientific-advice/. Accessed: 1 July 2020.

[67] McGuiness A. Coronavirus Track and Trace system can work without app, minister says. Sky News. 21 May 2020. Available: https://news.sky.com/story/coronavirus-track-and-trace-system-can-work-without-app-minister-says-11991959. Accessed: 1 July 2020.

[68] Blenkov A. The UK plans to issue coronavirus ‘immunity passports’ so people can leave the lockdown early. Business Insider. 3 April 2020. Available: https://www.businessinsider.com/uk-plans-coronavirus-immunity-passports-so-brits-can-leave-lockdown-2020-4?r=US&IR=T. Accessed: 1 July 2020.

[69] Wakefield J. NHS app paves the way for immunity passports. BBC. 27 May 2020. Available: https://www.bbc.co.uk/news/technology-52807414. Accessed: 1 July 2020.

[70] Goodman J, Carmichael F. Coronavirus: contact-tracing rumours debunked. BBC News. 13 June 2020. Available: https://www.bbc.co.uk/news/53021722. Accessed: 1 July 2020.

[71] Turk V. How Dominic Cummings could ruin the UK’s coronavirus response. Wired. 28 May 2020. Available: https://www.wired.co.uk/article/dominic-cummings-coronavirus-lockdown. Accessed: 1 July 2020.

[72] Lomas N. UK privacy and security experts warn over coronavirus app mission creep. TechCrunch. 29 April 2020. Available: https://techcrunch.com/2020/04/29/uk-privacy-and-security-experts-warn-over-coronavirus-app-mission-creep/?ncid=txtlnkusaolp00000618. Accessed: 1 July 2020.

[73] Burgess M. Just how anonymous is the Covid-19 contact tracing app? Wired. 12 May 2020. https://www.wired.co.uk/article/nhs-covid-app-data-anonymous. Accessed: 1 July 2020.

[74] Hall K. Pandemic, panic and patient data: tech firms and the NHS in a time of crisis. Prospect. 7 May 2020. Available: https://www.prospectmagazine.co.uk/politics/pandemic-panic-and-patient-data-tech-firms-and-the-nhs-in-a-time-of-crisis. Accessed: 1 July 2020.

[75] Privacy International. NHS deal with Palantir raises fears of vendor lock-in. Available: https://privacyinternational.org/examples/3814/nhs-deal-palantir-raises-fears-vendor-lock. Accessed: 1 July 2020.

[76] Fitzgerald M, Crider C. Under pressure, UK government releases NHS COVID data deals with big tech. Open Democracy. 5 June 2020. Available: https://www.opendemocracy.net/en/under-pressure-uk-government-releases-nhs-covid-data-deals-big-tech/. Accessed: 1 July 2020.

[77] Dearden. NHS coronavirus contact tracing app will have ‘unintended consequences’ senior official says. Independent. 4 May 2020. Available: https://www.independent.co.uk/news/health/nhs-coronavirus-tracing-app-nhsx-matthew-gould-a9498346.html. Accessed: 1 July 2020.

[78] Starkbaum J Felt U Negotiating the re-use of health data: Research, Big Data and the European General Data Protection Regulation. Big Data Soc. 2019 . 10.1177/2053951719862594

[79] Usher Institute. Statement on COVID-19 Symptom Tracker app. 7 April 2020. Available: https://www.ed.ac.uk/usher/breathe/latest/statement-covid-19-symptom-tracker-app. Accessed: 1 July 2020.

[80] Academy of Medical Sciences. Personal data for public good: using health information in medical research. 2006. Available: https://acmedsci.ac.uk/policy/policy-projects/personal-data. Accessed: 1 July 2020.

[81] Menni C Valdes AM Freidin MB Sudre CH Nguyen LH Drew DA Real-time tracking of self-reported symptoms to predict potential COVID-19. Nat Med. 2020. Online ahead of print. 10.1038/s41591-020-0916-2 32393804 PMC7751267

[82] Ministry of Defence. jHub support NHSX to securely share COVID-19 symptom data. UK Government. 19 May 2020. Available: https://www.gov.uk/government/news/jhub-support-nhsx-to-securely-share-covid-19-symptom-data. Accessed: 1 July 2020.

[83] Magrabi F Habli I Sujan M Wong D Thimbleby H Baker M Why is it so difficult to govern mobile apps in healthcare? BMJ Health Care Inform. 2019;26:e100006. 10.1136/bmjhci-2019-100006 31744843 PMC7252987

[84] Du Preez D. The UK does not need a new AI regulator, but the government is failing on openness. Diginomica/Government. 10 February 2020. Available: https://diginomica.com/uk-does-not-need-new-ai-regulator-government-failing-openness. Accessed: 1 July 2020.

[85] Singaporeans trust up in government and the media. Straits Times. 18 March 2019. Available: https://www.straitstimes.com/singapore/singaporeans-trust-up-in-govt-media-survey. Accessed: 1 July 2020.

[86] Sim D. Why aren’t Singapore residents using the TraceTogether contact tracing app? SCMP. 18 May 2020. Available: https://www.scmp.com/week-asia/people/article/3084903/coronavirus-why-arent-singapore-residents-using-tracetogether. Accessed: 1 July 2020.

[87] Patten T. A healthy dose of caution: an analysis of Australia’s My Health Record. Baker McKenzie. 17 April 2019. Avaiable: https://www.bakermckenzie.com/en/insight/publications/2019/04/a-healthy-dose-of-caution. Accessed: 1 July 2020.

[88] Barbaschow A. COVIDSafe legislation enters Parliament, with a few added privacy safeguards. ZDNet. 13 May 2020. Available: https://www.zdnet.com/article/covidsafe-legislation-enters-parliament-with-a-few-added-privacy-safeguards/. Accessed: 1 July 2020.

[89] University of Western Australia. Why only half of Australians downloaded the COVIDSafe App. Medical Xpress. 4 June 2020. Available: https://medicalxpress.com/news/2020-06-australians-downloaded-covidsafe-app.html. Accessed: 1 July 2020.

[90] Cooke H. Public surge of trust in government during Covid-19 crisis. Stuff. 8 April 2020. Available: 8^th^ 2020 https://www.stuff.co.nz/national/health/coronavirus/120911320/coronavirus-public-surge-in-trust-of-government-and-national-pride. Accessed: 1 July 2020.

[91] Bhatia R. Not nearly enough Covid-19 tracker app downloads, tech expert says. Stuff. 25 May 2020. Available: https://www.stuff.co.nz/national/health/coronavirus/300020263/coronavirus-not-nearly-enough-covid19-tracker-app-downloads-tech-expert-says. Accessed: 1 July 2020.

[92] Vezich D. Growing pressure for kiwis to use COVID-19 Tracer app amid new cases. News Hub. 18 June 2020. Available: https://www.newshub.co.nz/home/new-zealand/2020/06/growing-pressure-for-kiwis-to-use-covid-19-tracer-app-amid-new-cases.html. Accessed: 1 July 2020.

[93] Pagliari C. Privacy, data science and personalised medicine. Time for a balanced discussion. LinkedIn. 26 March 2015. Available: https://www.linkedin.com/pulse/privacy-data-science-personalised-medicine-time-claudia-pagliari/. Accessed: 1 July 2020.

[94] United Nations Regional Information Centre for Western Europe. Big Brother knows and still Icelanders are happy. UN website. 23 April 2020. Available: https://unric.org/en/big-brother-knows-and-still-icelanders-are-happy/. Accessed: 1 July 2020.

[95] Singapore Government. COVID-19: update of the latest confirmed cases in Singapore. 16 June 2020. Available: https://www.gov.sg/article/covid-19-cases-in-singapore. Accessed: 1 July 2020.

[96] Yu E. Singapore’s move to introduce wearable devices for contact tracing sparks public outcry. ZDNet. 7 June 2020. Available; https://www.zdnet.com/article/singapores-move-to-introduce-wearable-devices-for-contact-tracing-sparks-public-outcry/. Accessed: 1 July 2020.

[97] Taylor J. How did the CovidSafe app go from being vital to almost irrelevant? Guardian. 23 May 2020. Available: https://www.theguardian.com/world/2020/may/24/how-did-the-covidsafe-app-go-from-being-vital-to-almost-irrelevant. Accessed: 1 July 2020.

[98] Taylor J. Australia’s Covidsafe coronavirus app works less than one in four times for some devices. The Guardian. 17 June 2020. Available: https://www.theguardian.com/australia-news/2020/jun/17/covid-safe-app-australia-covidsafe-contact-tracing-australian-government-covid19-tracking-problems-working. Accessed: 1 July 2020.

[99] Kolbert E How Iceland beat the coronavirus. New Yorker. 2020;1 https://www.newyorker.com/magazine/2020/06/08/how-iceland-beat-the-coronavirus. Accessed: 1 July, 2020.

[100] Johnson B. MIT Technology Review. 11 May 2020. Available: https://www.technologyreview.com/2020/05/11/1001541/iceland-rakning-c19-covid-contact-tracing/. Accessed: 1 July 2020.

[101] Cousins S New Zealand eliminates COVID-19. Lancet. 2020;395:1474. 10.1016/S0140-6736(20)31097-7 32386582 PMC7252131

[102] Graham-McLay C. New Zealand puts Covid-19 quarantine in hands of military after border fiasco. Guardian. 17 June 2020. Available: https://www.theguardian.com/world/2020/jun/17/new-zealand-brings-in-military-after-covid-19-quarantine-fiasco. Accessed: 1 July 2020.

[103] Mansell R. Coronavirus contact tracing apps – a proportionate response? LSE Blogs. 23 April 2020. Available: https://blogs.lse.ac.uk/medialse/2020/04/23/coronavirus-contact-tracing-apps-a-proportionate-response/. Accessed: 1 July 2020.

[104] Scott M, et al. How Google and Apple outflanked governments in the race to build coronavirus apps. Politico, May 15^th^ 2020. Available: https://www.politico.eu/article/google-apple-coronavirus-app-privacy-uk-france-germany/. Accessed: 1 July 2020

[105] Bacohido. Privacy advocates point to flaws in the Apple-Google Covid-19 tracing app. Security Boulevard. 29 May 2020. Available: https://securityboulevard.com/2020/05/my-take-technologists-privacy-advocates-point-to-flaws-in-the-apple-google-covid-19-tracing-app/. Accessed: 1 July 2020.

[106] Privacy International. In big tech we trust: is Apple’s and Google’s COVID-19 reputation laundering enough to make us forget about their past? 22 May 2020. Available: https://privacyinternational.org/news-analysis/3843/big-tech-we-trust-apples-and-googles-covid-19-reputation-laundering-enough-make. Accessed: 1 July 2020.

[107] Ilves E. Why are Apple and Google dictating how European democracies fight coronavirus? The Guardian. 16 June 2020. Available: https://www.theguardian.com/commentisfree/2020/jun/16/google-apple-dictating-european-democracies-coronavirus. Accessed: 1 July 2020.

[108] Coronavirus: Health minister says app should roll out by winter. BBC News. 17 June 2020. Available: https://www.bbc.co.uk/news/technology-53083340. Accessed: 1 July 2020.

[109] Crouch H Tory peer reveals NHS contact tracing app has cost £11.8M to date. Digit Health. 2020;23 https://www.digitalhealth.net/2020/06/nhs-contact-tracing-app-cost/. Accessed July 1, 2020.

[110] Downey A NHSX knew contact tracing apps wouldn’t work on iPhones in April. Digit Health. 2020;25 https://www.digitalhealth.net/2020/06/nhsx-knew-contact-tracing-app-wouldnt-work-on-iphones-in-april/. Accessed July 1, 2020.

[111] Merrick J. Matt Hancock accuses Apple of “intransigence” in working with governments after u-turn over tracking app. iNews. 20 June 2020. Available: https://inews.co.uk/news/politics/matt-hancock-apple-intransigence-working-governments-u-turn-tracking-app-452028. Accessed: 1 July 2020.

[112] Germany launches coronavirus app to immediate criticism. Financial Times. 16 June 2020. Available: https://www.ft.com/content/70545c00-019c-499f-9bec-b52c9308e5a8. Accessed: 1 July 2020.

[113] O’Halloran J. Minister downgrades status of UK contact tracing app from vital to ‘cherry on cake’. Computer Weekly, June 12, 2012 https://www.computerweekly.com/news/252484581/Minister-downgrades-status-of-UK-contact-tracing-app-from-vital-to-cherry-on-cake. Accessed: 1 July 2020.

[114] Deputy First Minister. Coronavirus (COVID-19) strategic framework for re-opening schools, early learning and childcare provision. Scottish Government. 21 May 2020. Available: https://www.gov.scot/publications/excellent-equity-during-covid-19-pandemic-strategic-framework-reopening-schools-early-learning-childcare-provision-scotland/pages/3/. Accessed: 1 July 2020.

[115] Towards a Robust, Resilient, Wellbeing Economy for Scotland: Report of the Advisory Group on Economic Recovery. Scottish Government. 22 June 2020. Available: https://www.gov.scot/publications/towards-robust-resilient-wellbeing-economy-scotland-report-advisory-group-economic-recovery/. Accessed: 1 July 2020.

[116] NERVTAG. Key issues relating to contact tracing. Public Health England. 23 April 2020. Available: https://assets.publishing.service.gov.uk/government/uploads/system/uploads/attachment_data/file/890187/s0275-key-issues-for-contact-tracing-for-consideration-by-nervtag-230420-sage30.pdf. Accessed: 1 July 2020.

[117] Owen G, et al. Coronavirus track-and-trace army of 25,000 recruits will go into action this week - if the ‘world-beating’ app's glitches can be solved. Mail Online 23 May 2020. Available: https://www.dailymail.co.uk/news/article-8351291/COVID-19-track-trace-army-25-000-tracers-launch-week-apps-glitches-fixed.html. Accessed: 1 July 2020.

[118] Scottish Government. Coronavirus tourism and hospitality sector guidance. Available: https://www.gov.scot/publications/coronavirus-covid-19-tourism-and-hospitality-sector-guidance/pages/collecting-customer-contact-details/. Accessed: 1 July 2020.

[119] Haskins C, et al. We need an army of contact tracers to reopen the country. We might get apps instead. Buzzfeed 29 April 2020. Available: https://www.buzzfeednews.com/article/carolinehaskins1/coronavirus-contact-tracing-google-apple. Accessed: 1 July 2020

[120] Scottish Government. Protecting Scotland’s Future. 3 September 2019. Available: https://www.gov.scot/publications/protecting-scotlands-future-governments-programme-scotland-2019-20/pages/6/. Accessed: 1 July 2020.

[121] Scottish Government. Advisory board on social renewal: focus on equality, human rights and social justice. June 2020. Available: https://www.gov.scot/news/advisory-board-on-social-renewal/. Accessed: 1 July 2020.

