# Supplementary material for: The ethics and value of contact tracing apps: International insights and implications for Scotland’s COVID-19 response
Source: J Glob Health. 2020 Jul 21;10(2):020103. doi: 10.7189/jogh.10.020103 (PMC7510337; doi:10.7189/jogh.10.020103)
Supplement: Online Supplementary Document [file jogh-10.020103-s001.pdf]

Appendix S1. Brief Explainer – Centralised vs Decentralised Proximity Tracking

Unlike location tracking, proximity monitoring uses Bluetooth Low Energy beacons to exchange encrypted ‘keys’ between nearby devices, which are logged as digital ‘handshakes’

Centralised: Users are allocated an anonymised identity code when registering, which is stored on a central database. If someone tests positive for Covid-19 and contacts the public health authority, information about both parties in the handshake will be uploaded to the database and cross referenced with the stored identities. If they match, an alert will be sent to the relevant phone along with instructions to self-isolate and contact the health authority if symptoms worsen.

Fully decentralised: Proximity handshakes are kept on users’ smartphones. If a user tests positive, and chooses to alert others, their anonymised identifier only is sent to the mobile provider’s database, which all phones have access to. Contact matching and alert sending is done on phones themselves, rather than by the central authority. Both parties remain anonymous. All users receive is an ‘exposure notification’.

Semi-decentralised: As with decentralised, but mediated via a government-approved app, which can generate advice and contact information along with the alert. In practice most of the ‘decentralised’ approaches used in different countries use a version of this hybrid approach.

Fully decentralised models offer the most protection from government surveillance but minimal value for users (sparse information) or public health authorities (depends entirely on how users respond to the alert). Centralisation opens up possibilities to produce ‘social graphs’ or to link data for health intelligence, research and innovation, but risks privacy and mission creep. Semi-centralised, choice-based, privacy-protecting models offer a compromise. All of these methods are imperfect, however – Bluetooth is hackable and may falsely log people separated by walls or windows, and while proximity notification is theoretically possible without an app, it is typically part of one, which may be connected to other apps and databases, representing different layers of security and privacy risk.

Articles about contact tracing apps often refer to the Apple-Google approach. This is an application programming interface (API) which can make it easier for approved government apps to communicate with both iPhones and Android devices. It can run in the background, while phones are ‘asleep’, thus logging handshakes while conserving battery life. It is ‘decentralised’ because data and matching happens on phones, and ‘privacy protecting’ because identifiers are anonymised, and it limits what data may be collected by the apps. In a forthcoming update, this will be embedded into the Apple and Android operating systems, meaning that no app will technically be needed for handshakes to be exchanged, although users will still need to download one to either declare themselves as Covid-19 positive or to learn if someone they have come into contact with was diagnosed.

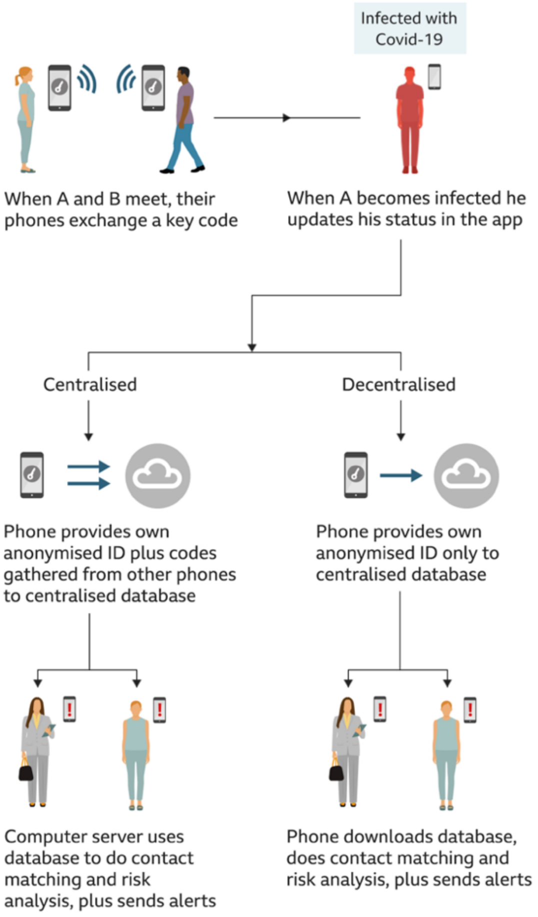

Infographic from https://www.bbc.co.uk/news/technology-52355028

N.B. There are various ways of characterising these different approaches, from simple infographics to sophisticated analyses of the cryptography underlying them. These lay descriptions are offered as an optional accompaniment to the paper but numerous alternatives are available online.
